# Geriatric Rheumatology: The Need for a Separate Subspecialty in the Near Future

**DOI:** 10.7759/cureus.8474

**Published:** 2020-06-06

**Authors:** Pooja Poudel, Jianghong Yu

**Affiliations:** 1 Internal Medicine, State University of New York Upstate Medical University, Syracuse, USA; 2 Rheumatology, State University of New York Upstate Medical University, Syracuse, USA

**Keywords:** geriatric rheumatology, gerontorheumatology

## Abstract

Rheumatology is a broad specialty in itself, and it involves caring for patients of all age groups. Patients of different age groups have different characteristics and a one-size-fits-all approach is not feasible in catering to their diverse medical needs. The presentation and the manifestations of diseases vary in different age groups. We have pediatric rheumatology as a separate subspecialty where pediatric patients with rheumatological diseases are provided specific care best suited to their needs. However, for older patients, such a separate subspecialty is not widely available in medical practice. Geriatric rheumatology or gerontorheumatology is a branch of rheumatology dealing with older patients with rheumatological diseases. It is high time to consider establishing geriatric rheumatology as a separate subspecialty to provide better care for older patients.

## Introduction and background

Evaluation of elderly patients is often medically challenging because of the co-morbid conditions, limited social support, and polypharmacy. Geriatric rheumatology or gerontorheumatology is a branch of medicine that deals with joints, muscles, and connective tissues in the elderly population [1]. We need to have patients' compliance and involvement in diagnosing and managing rheumatological diseases accurately, which is often challenging because of the cognitive decline and lack of motivation and social support in the older population. Musculoskeletal disorders are most common in the elderly population. As the number of people over 65 years is increasing day by day, it is estimated that the number of older patients with musculoskeletal problems will double in the coming decades. Hence, it will be very challenging for rheumatology to meet the healthcare needs of the growing elderly population. In this article, we discuss the pattern of rheumatological disease in the elderly, the challenges faced in meeting their healthcare demands, and the things rheumatologists need to keep in mind while taking care of elderly patients with rheumatological diseases.

## Review

Age-related changes in the immune system and pattern of rheumatological diseases

The immune system undergoes changes as we age and it is necessary to describe the disease course of rheumatic diseases in the elderly. It is important to understand the immune senescence and the age-related changes in determining the disease course in older rheumatology patients.

The clearance of dead cells from the body is important in the maintenance of the immune system function. Disruption of the apoptotic process can result in the accumulation of dead cells and lead to the inflammatory process. The clearance of the apoptotic cells was recently observed to be reduced in aged mice [2]. Aging is also known to be associated with a decreased ability to clear apoptotic cells. This uncleared debris acts as a potential source of auto-antigens, which leads to the formation of autoantibodies, further leading to autoimmunity [3].

The immune system becomes less effective at producing and activating T and B cells as we age. There is a loss of regenerative capacity and defects appear in T and B cell production, maturation, and function. This makes it more prone to intolerance to the pro-inflammatory environment. Along with the loss of effectiveness of the adaptive immune system, immunosenescence is also characterized by an increasing incidence of autoimmune disorders [4]. Alterations in the innate immune system cause monocyte and macrophage activation, which causes the release of inflammatory markers. The poor inflammatory environment may lead to the development of self-reactive T and B cells and promote the development of inflammatory diseases like rheumatoid arthritis (RA).

Human beings are chronically exposed to stress, both mental and physical. The relationship between stress response and aging pathways has been observed in invertebrates but clear evidence in humans is lacking [5]. Interestingly, it has been observed that the telomere length at birth is affected by prenatal stress [6]. Also, it would be interesting to find the relationship between stress and leukocyte telomere shortness especially since telomere shortening in chondrocytes and leucocytes have been observed in osteoarthritis [7,8].

Epigenetic mechanisms (DNA methylation, post-translational histone code, etc) are known to influence the pathogenesis of systemic lupus erythematosus (SLE), RA, scleroderma, and osteoarthritis [9]. Age-related epigenetic changes influence aging and are linked to abnormal T-cell function, which in turn may contribute to a high incidence of autoimmunity in old age. The possibility of reversal of aging and slowing the progression of the rheumatological disease by targeting epigenetic mechanisms is an exciting area of research and may lead to newer treatment modalities for age-related rheumatological diseases. Also, senescent cells produce proteins known as senescence-associated secretory phenotypes (SASPs), which are known to contribute to chronic inflammation in aging [10]. Senolytics, drugs targeting the removal of senescent cells to slow aging, have been tested in animals [11]. However, the effectiveness of this drug in humans and rheumatological disease is unclear.

Inflammation

Aging is a pro-inflammatory state, a fact that has been confirmed by studies where an elevated level of pro-inflammatory cytokines is noted in healthy older adults compared to younger adults. The term “Inflamm-aging” refers to a chronic low-grade inflammatory response to the chronic antigenic burden. The term was first coined when Fagiolo et al. noted that the serum from older people was found to have produced a higher amount of cytokines than that of younger people [12]. Inflamm-aging is different from acute inflammation, which refers to an acute immunologic response to injury. IL-6, one of the SASPs, has been associated with rheumatological disease including RA, osteoarthritis, polymyalgia rheumatica, and giant cell arteritis [13].

Macromolecular Damage/Oxidative Stress Injury

According to Harman's free radical theory of aging (FRTA), oxidative damage to the DNA and other cellular components occur over time and lead to aging and disease [14]. Oxidative damage can cause DNA damage, which contributes to the induction and flare of autoimmune diseases such as SLE and RA.

Bone Aging

Resorption and formation of bone occur all the time. Bone continuously undergoes the remodeling process. There is net bone formation during adolescence. The rate of bone growth ceases and then stops fully when peak bone mass is achieved, which usually occurs by the age of 15-25 years [15]. The total bone mass then begins to decrease by the third and fourth decade of life, and the estimated bone mass will be 50% of its peak value by the age of 80. This process is known as senile osteoporosis. Women undergo an accelerated period of bone loss due to menopause. Menopause is a state of estrogen deficiency. The exact cellular mechanism by which estrogen deficiency accelerates bone turnover is not known. However, it has been shown that increased cytokine production plays a role in promoting osteoclast production in the estrogen-deficient state. Many inflammatory markers like IL-6 and TNF alpha increase during the aging process. These are the promoters of osteoclast differentiation and activation and thus cause net bone resorption [16]. The use of mesenchymal stem cells in osteoarthritis treatment has been one of the highlights of research in recent years [17]. However, the safety and effectiveness of these cells need to be evaluated and investigated further. We need more clinical trials in the future for establishing the effectiveness of mesenchymal stem cells.

The Pattern of Rheumatological Diseases

Certain rheumatological conditions such as polymyalgia rheumatica, osteoporosis, pseudogout, and osteoarthritis occur in the latter half of life; while other conditions such as rheumatoid arthritis, ankylosing spondylitis manifest at a younger age, their clinical manifestations may exacerbate with advancing age [18]. The incidence gout among the older patient population has increased due to renal impairment, diuretic use, hypertension, and metabolic syndrome [19,20]. The manifestation of late-onset gout is almost similar to early-onset gout though the episodes are known to occur frequently in older patients.

The peak incidence of RA occurs between the age of 35-55 years. Late-onset rheumatoid arthritis (LORA) occurs usually after the age of 60 years. LORA usually has large-joint involvement. Complications of RA are less common in LORA compared to early-onset rheumatoid arthritis (EORA), which include rheumatoid hand deformities, cardiovascular disease, and chronic kidney disease. Laboratory markers such as rheumatoid factor and anti-cyclic citrullinated peptide (anti-CCP) occur less frequently in patients with LORA compared to EORA patients [21].

Similarly, late-onset SLE is known to have different antibody profiles compared to early-onset SLE. It is characterized by a lower frequency of anti-RNP antibody, anti-Sm antibody, variable frequency of anti dsDNA antibodies, and higher frequency of rheumatoid factor, anti-Ro, and anti-La antibodies [22]. Compared to early-onset SLE, patients usually present with non-specific symptoms, and diagnosis is often difficult as it mimics aging symptoms. However, the long-term prognosis is not good. Late-onset SLE tends to exhibit more cardiovascular and renal complications and more chances of malignancy even though the clinical presentation appears to be mild [23]. The older patient population has a higher incidence of drug-induced lupus due to the frequent use of medications like statins, thiazides, and proton pump inhibitors [24,25].

Overall, rheumatic diseases of late-onset exhibit different clinical and immunological profiles compared to those of early-onset, which can lead to underdiagnosis of the disease. Even though the disease sometimes appears to have a mild clinical presentation, complications and prognosis can be bad due to co-morbidities in the old age [26]. It is necessary to carefully assess elderly patients presenting with rheumatic symptoms so that the disease does not go underdiagnosed or missed.

Challenges in the diagnosis of rheumatological diseases and their management

Disease manifestations of rheumatological diseases may cause other diagnostic problems. The rheumatologist has to consider a broad range of differentials and treatment possibilities. Both the pharmacological and nonpharmacological interventions that are appropriate in regular treatment may be less appropriate in older patients. Older patients are more vulnerable to the side effects of the medications. Clinicians should expect an age-related gradual reduction in renal function. Pharmacokinetics and pharmacodynamics are affected by age-related changes in metabolism. Older patients have greater co-morbidities and they are often on multiple medications, which increases the risk of adverse events due to drug interactions. Mode of administration of the drug might change in the advanced age due to eyesight and hand dexterity issues. For example, it is more feasible to administer infusions to older patients instead of providing self-injectables. Additionally, intellectual and cognitive functions in older patients are impaired, which affects their ability to communicate their symptoms. Finally, compliance with medications is a huge issue in older patients, which is harder to improve especially if they have cognitive impairment. Geriatric patients usually suffer from functional impairments, and we need to develop the best possible ways to provide optimal care. Medications need to be minimized and patients need to follow up with primary care physician regularly. Advance care directives need to be set up in a timely manner.

The Department of Rheumatology at the Sint Maartenskliniek hospital in Nijmegen, Netherlands, has established a gerontorheumatological outpatient service to adequately meet the needs of the aging population [27]. This specialty aims to improve the quality of life of the older population by preventing unnecessary disabilities. The gerontorheumatological service differs from the regular rheumatological service in that it uses a holistic approach in the management of the diseases by taking into account age-related psychological, social, and cultural issues.

With a gerontorheumatological outpatient service, the patient can be scheduled with a rheumatologist and a specialized nurse practitioner in geriatrics. The rheumatologist would focus on the rheumatological issues and the nurse practitioner would evaluate the patient’s cognitive functioning, functional activities of daily living, and the social environment of the patient’s residence. Then, directly after the patient encounter, the rheumatologist and the nurse practitioner can decide about the future plan of care.

A rheumatologist can contribute in many ways in the management of the geriatric patient population. For example, rheumatologists are not generally involved in the management of fractured hips and are omitted from the long-term management. Hip fracture in the elderly is associated with a 44% rate of mortality within one year after the event. Most of it is caused due to the patient’s failure to regain mobility. A rheumatologist can make a great contribution to improving this as they are experts in musculoskeletal disease and maintenance of mobility. Also, under-treatment of osteoporosis in older patients is a huge issue [28]. Patients with osteoporosis are rarely referred to a rheumatologist. There is much more that can be done through exercise, postural assistance, and local injections with the help and advice of a rheumatologist.

Elderly patients are less familiar with the internet and technology. A lack of or restricted access to online resources creates a barrier for them to interact with online patient groups. We need to put in place more measures to ease the path for appropriate communication with the patients and adapt to the new modes of drug administration in the elderly.

## Conclusions

With a rapidly growing older patient population and their increasing co-morbidities, managing rheumatological disease in them is often challenging. Establishing a well-structured outpatient service for these patients where we can address both their geriatric and rheumatological problems would be immensely beneficial and productive for them as well as the providers. We need to think about establishing more gerontorheumatologic outpatient services in the coming days to address the challenges in caring for the elderly and to improve their quality of life.

## References

[1] van Lankveld W, Franssen M, Stenger A (2005). Gerontorheumatology: the challenge to meet health-care demands for the elderly with musculoskeletal conditions. Rheumatology (Oxford).

[2] Aprahamian T, Takemura Y, Goukassian D, Walsh K (2008). Ageing is associated with diminished apoptotic cell clearance in vivo. Clin Exp Immunol.

[3] Cohen PL, Caricchio R, Abraham V (2002). Delayed apoptotic cell clearance and lupus-like autoimmunity in mice lacking the c-mer membrane tyrosine kinase. J Exp Med.

[4] Ray D, Yung R (2018). Immune senescence, epigenetics and autoimmunity. Clin Immunol.

[5] Lithgow GJ, Miller RA (2008). Determination of aging rate by coordinated resistance to multiple forms of stress. Molecular Biology of Aging.

[6] Price JD, Beauchamp NM, Rahir G, Zhao Y, Rieger CC, Lau-Kilby AW, Tarbell KV (2014). CD8+ dendritic cell-mediated tolerance of autoreactive CD4+ T cells is deficient in NOD mice and can be corrected by blocking CD40L. J Leukoc Biol.

[7] Price JS, Waters JG, Darrah C, Pennington C, Edwards DR, Donell ST, Clark IM (2002). The role of chondrocyte senescence in osteoarthritis. Aging Cell.

[8] Zhai G, Aviv A, Hunter DJ (2006). Reduction of leucocyte telomere length in radiographic hand osteoarthritis: a population-based study. Ann Rheum Dis.

[9] Boddaert J, Huong DL, Amoura Z, Wechsler B, Godeau P, Piette JC (2004). Late-onset systemic lupus erythematosus: a personal series of 47 patients and pooled analysis of 714 cases in the literature. Medicine (Baltimore).

[10] Coppé JP, Patil CK, Rodier F (2008). Senescence-associated secretory phenotypes reveal cell-nonautonomous functions of oncogenic RAS and the p53 tumor suppressor. PLoS Biol.

[11] Zhu Y, Tchkonia T, Pirtskhalava T (2015). The Achilles’ heel of senescent cells: from transcriptome to senolytic drugs. Aging Cell.

[12] Franceschi C (2007). Inflammaging as a major characteristic of old people: can it be prevented or cured?. Nutr Rev.

[13] Lasry A, Ben-Neriah Y (2015). Senescence-associated inflammatory responses: aging and cancer perspectives. Trends Immunol.

[14] Harman D (1956). Aging: a theory based on free radical and radiation chemistry. J Gerontol.

[15] Kong L, Zheng LZ, Qin L, Ho KKW (2017). Role of mesenchymal stem cells in osteoarthritis treatment. J Orthop Translat.

[16] Kloss FR, Gassner R (2006). Bone and aging: effects on the maxillofacial skeleton. Exp Gerontol.

[17] Bruunsgaard H (2002). Effects of tumor necrosis factor-alpha and interleukin-6 in elderly populations. Eur Cytokine Netw.

[18] Michet CJ Jr, Evans JM, Fleming KC, O'Duffy JD, Jurisson ML, Hunder GG (1995). Common rheumatologic diseases in elderly patients. Mayo Clin Proc.

[19] De Leonardis F, Govoni M, Colina M, Bruschi M, Trotta F (2007). Elderly-onset gout: a review. Rheumatol Int.

[20] Bolzetta F, Veronese N, Manzato E, Sergi G (2012). Tophaceous gout in the elderly: a clinical case review. Clin Rheumatol.

[21] Huscher D, Sengler C, Gromnica-Ihle E (2013). Clinical presentation, burden of disease and treatment in young-onset and late-onset rheumatoid arthritis: a matched-pairs analysis taking age and disease duration into account. Clin Exp Rheumatol.

[22] Koh ET, Boey ML (1994). Late onset lupus: a clinical and immunological study in a predominantly Chinese population. J Rheumatol.

[23] Lin H, Wei JC, Tan CY (2012). Survival analysis of late-onset systemic lupus erythematosus: a cohort study in China. Clin Rheumatol.

[24] Borchers AT, Keen CL, Gershwin ME (2007). Drug-induced lupus. Ann NY Acad Sci.

[25] Vasoo S (2006). Drug-induced lupus: an update. Lupus.

[26] Dhaon P, Tripathy SR (2016). Rheumatic disease in elderly population, how different from the conventional presentations?. Internet J Rheumatol Clin Immunol.

[27] van Lankveld W, Goossens J, Franssen M (2011). The gerontorheumatology outpatient service: toward an international classification of function-based health care provision for the elderly with musculoskeletal conditions. Geriatric Rheumatology: A Comprehensive Approach.

[28] Johnell K, Fastbom J (2009). Undertreatment of osteoporosis in the oldest old? A nationwide study of over 700,000 older people. Arch Osteoporos.

